# Dietary phytochemical index is associated with systemic inflammation, insulin resistance, and gut microbiota-derived metabolites in individuals with obesity: A cross-sectional study

**DOI:** 10.1371/journal.pone.0347754

**Published:** 2026-04-27

**Authors:** Lingtao Zheng, Yanjiao Shen

**Affiliations:** 1 Department of Endocrinology, Xingtai People’s Hospital, Xingtai City, Hebei Province, China; 2 Tuberculosis Department One, Xingtai Fifth Hospital, Xingtai City, Hebei Province, China; Emory University School of Medicine, UNITED STATES OF AMERICA

## Abstract

**Background:**

Obesity is linked to chronic inflammation, insulin resistance, and gut microbiota dysbiosis, partly via TMAO. This study investigates associations between dietary patterns, as indexed by the Dietary Phytochemical Index (DPI), with insulin resistance, inflammatory markers, and TMAO.

**Methods:**

In this cross-sectional study, 600 adults with obesity (BMI ≥ 30 kg/m²) without diagnosed comorbidities were evaluated. Dietary intake was assessed using a validated 110-item FFQ, and the DPI was computed. Fasting blood samples were analyzed for glucose, insulin, HOMA-IR, lipid profile, CRP, IL-6, TNF-α, LBP, adiponectin, and TMAO. Multivariable regression and mediation analyses were performed.

**Results:**

Participants in the highest DPI quartile exhibited lower mean BMI, waist circumference, and fat mass compared to those in the lowest quartile. They also had lower fasting plasma glucose (4.52 ± 0.06 mmol/L vs. 5.92 ± 0.24 mmol/L; P < 0.001), lower insulin concentrations (65.6 ± 2.4 pmol/L vs. 120.4 ± 18.0 pmol/L; P < 0.001), and lower HOMA-IR values (1.90 ± 0.09 vs. 4.59 ± 0.82; P < 0.001). Higher DPI was associated with lower concentrations of CRP, TNF-α, and TMAO, and higher concentrations of adiponectin and HDL-C (all P < 0.001).

**Conclusions:**

Higher DPI was associated with lower systemic inflammation and better insulin sensitivity. These findings highlight the potential role of phytochemical-rich diets in supporting insulin resistance and metabolic disturbances among obese adults; however, prospective randomized controlled trials are required to confirm these associations and establish causality.

## Introduction

Obesity is a major global public health concern and a key risk factor for metabolic syndrome, insulin resistance (IR), and cardiovascular diseases [1,2]. Chronic low-grade inflammation in obesity contributes to insulin resistance, dyslipidemia, and cardiovascular risk [3,4]. Emerging evidence indicates that gut microbiota and its metabolites, such as Trimethylamine-N-oxide (TMAO), play a central role in mediating obesity-related metabolic and inflammatory disturbances [5]. Dysbiosis of gut microbiota, often observed in obese individuals, promotes increased intestinal permeability, endotoxemia, and systemic inflammation, which collectively exacerbate metabolic dysfunction [6].

Diet is a modifiable determinant of both inflammation and gut microbiota composition. Diets rich in plant-based foods provide high levels of bioactive compounds, including polyphenols, carotenoids, and flavonoids, collectively referred to as phytochemicals [7]. The Dietary Phytochemical Index (DPI), a validated metric reflecting the proportion of daily energy derived from phytochemical-rich foods, has been inversely associated with markers of inflammation, oxidative stress, and cardiometabolic risk [8,9]. Unlike indices that focus on single nutrients or food components—such as dietary fiber intake or the Dietary Inflammatory Index (DII), which primarily quantifies the inflammatory potential of nutrients—the DPI captures the overall contribution of phytochemical-rich foods to total energy intake [8]. This approach encompasses a broader spectrum of bioactive compounds including flavonoids, carotenoids, phenolic acids, and lignans that may exert synergistic effects beyond those attributable to fiber alone [9]. While high-fiber diets certainly confer health benefits, the DPI specifically quantifies the proportion of energy derived from foods naturally rich in phytochemicals, providing a more comprehensive assessment of plant-based dietary patterns and their potential cumulative effects on metabolic health [10]. Previous studies have shown that higher DPI scores are associated with reduced C-reactive protein (CRP), interleukin-6 (IL-6), and tumor necrosis factor-alpha (TNF-α), highlighting the potential for diet to modulate inflammatory pathways [10].

Few studies have comprehensively examined how phytochemical intake interacts with gut microbiota diversity and metabolites like TMAO to influence insulin resistance [11–13]. Moreover, most studies have focused on individual nutrients or single food groups, rather than comprehensive dietary patterns, limiting understanding of their collective effects on metabolic and microbial outcomes [14]. Understanding these complex interactions is crucial for developing dietary strategies to improve metabolic health in obese individuals.

The present study aims to investigate the associations between DPI, and systemic inflammation, insulin resistance, and TMAO levels in adults with obesity without diagnosed comorbidities. To our knowledge, this is among the first studies to integrate DPI, inflammatory and metabolic biomarkers, and microbial metabolites in a single comprehensive analysis, providing novel insights into the mechanisms linking diet quality to metabolic health. It has been hypothesized that a higher DPI improves metabolic health in individuals with obesity by modulating gut metabolites and reducing systemic inflammation.

## Methods

### Study design and participants

**Fig 1:**
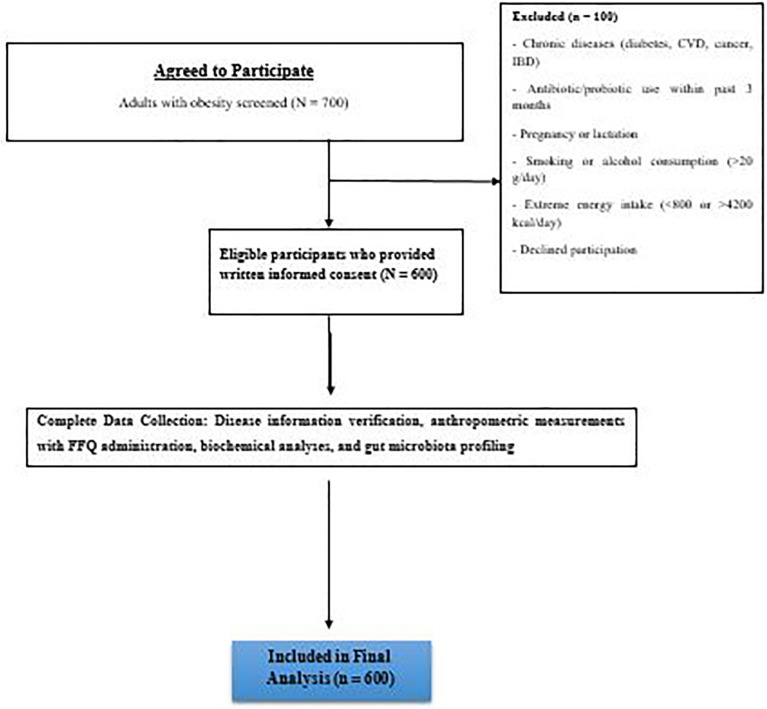
Flow diagram of participant recruitment and inclusion. A total of 700 adults with obesity were screened at Xingtai People’s Hospital between January and October 2025. Of these, 50 were excluded due to not meeting inclusion criteria (e.g., chronic diseases, recent antibiotic/probiotic use, pregnancy/lactation, extreme energy intake) or declining participation. The final analysis included 600 participants who provided written informed consent.

This cross-sectional study was conducted between 01/01/2025 and 31/10/2025 among adults with obesity without diagnosed comorbidities recruited from outpatient clinics in Xingtai People#39;s Hospital, China. Eligible participants were aged 18–55 years with a BMI ≥ 30 kg/m² and abdominal obesity. Exclusion criteria included history of chronic diseases (e.g., diabetes, cardiovascular disease, cancer, inflammatory bowel disease), use of antibiotics, probiotics, or prebiotics within the past 3 months, pregnancy or lactation, smoking, alcohol consumption (> 20 g/day), or extreme energy intake (< 800 or > 4200 kcal/day). Of 700 individuals screened, 600 met inclusion criteria and provided written informed consent (Fig 1). The study was approved by the Ethics Committee of Xingtai People’s Hospital (20241005−32) and conducted in accordance with the Declaration of Helsinki. The study followed STROBE guidelines for cross-sectional studies.

### Dietary assessment

Dietary intake over the previous year was evaluated using a validated 110-item semi-quantitative FFQ administered by trained dietitians [15]. Frequency and portion size of food items were assessed using household measures and food models. Daily nutrient intakes were calculated using the Chinese Food Composition Table, including total energy and macronutrients. Implausible values were excluded.

### Dietary phytochemical index (DPI)

The Dietary Phytochemical Index (DPI) was computed according to the method proposed by McCarty [8] with modifications for the Chinese dietary context:

DPI = (daily energy from phytochemical-rich foods in kcal ÷ total daily energy intake in kcal) × 100

The DPI was calculated as the percentage of total daily energy derived from phytochemical-rich foods, including fruits, vegetables, legumes, whole grains, nuts, and seeds. Energy adjustment was performed to reduce bias related to total caloric intake. Classification of phytochemical-rich foods: Foods were classified as phytochemical-rich based on established criteria and included: all fruits (fresh, dried, and 100% fruit juices without added sugar); all vegetables (excluding white potatoes due to their high glycemic load and lower phytochemical density relative to other vegetables); legumes (including soybeans and soy products); whole grains (defined as containing ≥25% whole grain content by weight); nuts and seeds; tea (all types); and coffee. Foods with added fats or sugars (e.g., fruit pies, sweetened beverages, candied fruits) were excluded even if they contained some phytochemicals, as their inclusion would not reflect the intended construct of phytochemical density relative to energy. Energy intake outliers (<800 kcal/day or >4200 kcal/day) were excluded prior to analysis (n = 18 screened participants). For the remaining sample, DPI values were examined for extreme values using boxplot analysis; no additional outliers were identified. DPI interpretation and quartile grouping: The DPI score represents the percentage of total daily energy derived from phytochemical-rich foods. Higher scores indicate greater relative contribution of these foods to overall energy intake. There is no established “normal range” for DPI in population studies, as values vary considerably across dietary patterns.

### Anthropometric and clinical measurements

Height was measured to the nearest 0.1 cm using a stadiometer, and weight to the nearest 0.1 kg using a digital scale (Seca, Germany). BMI was calculated as weight (kg) / height² (m²). Waist circumference was measured at the midpoint between the lower rib margin and iliac crest. Body composition (fat and muscle mass) was assessed via bioelectrical impedance analysis (InBody 770, Korea) under standardized conditions. Physical activity was assessed using the International Physical Activity Questionnaire (IPAQ) short form, expressed as MET-hours/week.

### Biochemical and inflammatory analyses

Fasting venous blood samples were collected after a 12-hour overnight fast between 08:00–10:00. Blood was collected into three types of tubes: (1) fluoride oxalate tubes for plasma glucose determination, processed immediately and centrifuged within 30 minutes to prevent glycolysis; (2) serum separator tubes for lipid profile and insulin assays, allowed to clot for 30 minutes at room temperature before centrifugation; and (3) EDTA tubes for inflammatory markers and TMAO, kept on ice and centrifuged within 2 hours. All samples were centrifuged at 1500 × g for 15 minutes at 4°C, aliquoted, and stored at −80°C until analysis. All biochemical measurements were conducted in certified clinical laboratories using standardized enzymatic and immunoassay methods. Internal quality control and calibration procedures were applied in accordance with laboratory guidelines.

Glycemic and lipid markers: Fasting plasma glucose was measured within 2 hours of collection using the hexokinase method on an automated chemistry analyzer (AU680, Beckman Coulter, USA). Serum insulin was measured by electrochemiluminescence immunoassay (ECLIA) on a Cobas e411 analyzer (Roche Diagnostics, Switzerland). Total cholesterol, HDL-cholesterol, and triglycerides were measured using enzymatic colorimetric methods on the AU680 analyzer. LDL-cholesterol was calculated using the Friedewald equation for samples with triglycerides <400 mg/dL, or measured directly by homogeneous enzymatic assay for samples exceeding this threshold. Insulin resistance was estimated using the Homeostatic Model Assessment for Insulin Resistance (HOMA-IR): HOMA-IR = (fasting plasma glucose in mmol/L × fasting insulin in μIU/mL) ÷ 22.5.

Inflammatory markers: High-sensitivity C-reactive protein (hs-CRP), interleukin-6 (IL-6), tumor necrosis factor-alpha (TNF-α), lipopolysaccharide-binding protein (LBP), and adiponectin were measured in serum using enzyme-linked immunosorbent assay (ELISA) kits (R&D Systems, USA) according to the manufacturer#39;s protocols. All samples were assayed in duplicate. Intra- and inter-assay coefficients of variation were <5% and <10%, respectively.

Gut microbiota metabolite: Plasma TMAO was quantified using ultra-performance liquid chromatography-tandem mass spectrometry (UPLC-MS/MS) (Acquity UPLC/Xevo TQ-S, Waters, USA). The limit of detection was 0.1 μM, and the calibration curve demonstrated R² > 0.99 across the analytical range.

### Statistical analysis

Sample size powered to detect medium effect size (f = 0.25) across quartiles with α = 0.05, power = 0.80, requiring n = 150 per group (G*Power 3.1). Two-sided p < 0.05 indicated significance. Continuous variables are mean ± SD or median (IQR), categorical variables as n (%). Normality of continuous variables was assessed using the Shapiro-Wilk test and visual inspection of Q-Q plots. Variables with approximately normal distribution are presented as mean ± standard deviation and were compared using one-way ANOVA. Variables with skewed distribution are presented as median (interquartile range) and were compared using Kruskal-Wallis tests with post-hoc Dunn#39;s tests for pairwise comparisons. Differences across DPI quartiles were analyzed with one-way ANOVA or Kruskal-Wallis tests; post-hoc Bonferroni correction applied. Multivariable linear regression examined associations between DPI quartiles (independent variable) and outcomes (inflammatory markers, and metabolic parameters), adjusting for age, sex, BMI, physical activity, energy, and fiber intake.

## Results

A total of 600 participants were included in the analysis. Table 1 presents the demographic, anthropometric, and dietary characteristics across quartiles of the DPI. The mean DPI in the cohort was 29.24 ± 10.51, increasing progressively from Q1 (15.18 ± 3.18) to Q4 (41.69 ± 2.40; P < 0.001). Age, marital status, and education level did not differ significantly across quartiles, while sex distribution showed a non-significant trend (P = 0.083). Notably, higher DPI was associated with lower BMI, waist circumference, and fat mass. muscle mass was lower in higher DPI quartiles, likely reflecting overall lower total energy intake in this group. Participants in higher DPI quartiles also reported lower total energy, carbohydrate, protein, and fat intake (all P < 0.001), suggesting a dietary pattern enriched in phytochemicals and lower in energy-dense foods.

**Table 1:** Demographic, Anthropometric, and Dietary Intake Characteristics by DPI Quartiles (N = 600).

Variables	Total (N = 600)	Q1 (n = 164)	Q2 (n = 129)	Q3 (n = 151)	Q4 (n = 156)	P value
**DPI**	29.24 ± 10.51	15.18 ± 3.18	25.97 ± 2.94	34.39 ± 1.65	41.69 ± 2.40	<0.001
**Age (years)**	38.33 ± 3.95	38.74 ± 5.45	38.61 ± 3.17	37.59 ± 4.24	38.37 ± 1.82	0.279
**Sex (male/female)**	139/161	33 / 49	25 / 39	36 / 40	45 / 33	0.083
**Marital status (single/married)**	149/151	37 / 45	28 / 36	44 / 32	40 / 38	0.294
**Education level (1 / 2 / 3)**	19/107/87	7 / 51 / 24	6 / 36 / 22	5 / 51 / 20	1 / 56 / 21	0.310
**Smoking (yes/no)**	91/209	29 / 53	23 / 41	18 / 58	21 / 57	0.267
**Alcohol consumption (yes/no)**	76/224	29 / 53	14 / 50	17 / 59	16 / 62	0.108
**BMI (kg/m²)**	32.31 ± 1.70	34.51 ± 0.79	32.53 ± 1.04	31.71 ± 0.52	30.41 ± 0.47	<0.001
**WC (cm)**	97.81 ± 6.03	105.63 ± 2.63	98.58 ± 4.56	95.22 ± 1.50	91.46 ± 1.19	<0.001
**Fat mass (%)**	37.63 ± 3.12	41.62 ± 1.39	38.35 ± 1.85	36.31 ± 0.72	34.11 ± 0.90	<0.001
**Muscle mass (%)**	33.98 ± 2.40	36.42 ± 2.24	35.12 ± 1.41	32.69 ± 0.70	31.74 ± 0.89	<0.001
**Energy intake (kcal/day)**	2314.8 ± 171.8	2531.6 ± 79.5	2323.8 ± 151.9	2256.7 ± 36.4	2136.2 ± 45.0	<0.001
**Carbohydrate (g/day)**	289.35 ± 21.48	316.45 ± 9.94	290.47 ± 18.99	282.09 ± 4.55	267.02 ± 5.63	<0.001
**Protein (g/day)**	104.17 ± 7.73	113.92 ± 3.58	104.57 ± 6.84	101.55 ± 1.64	96.13 ± 2.03	<0.001
**Fat (g/day)**	82.30 ± 6.11	90.01 ± 2.83	82.62 ± 5.40	80.24 ± 1.29	75.95 ± 1.60	<0.001

Values are presented as mean ± standard deviation for continuous variables and as number of participants (n) for categorical variables. P values are based on one-way ANOVA for continuous variables and Pearson’s Chi-Square test for categorical variables across quartiles of the Dietary Phytochemical Index (DPI). Education levels: 1 = primary or less, 2 = secondary, 3 = university or higher. Q1 = lowest, Q4 = highest DPI quartile.

Table 2 summarizes inflammatory markers and gut metabolites profiles by DPI quartiles. Participants with higher DPI had significantly lower systemic inflammation, evidenced by decreased CRP, IL-6, TNF-α, TMAO, and LBP (all P < 0.001), and higher anti-inflammatory adiponectin levels.

**Table 2:** Metabolic, Inflammatory, and Gut Metabolites Biomarkers by DPI Quartiles.

Variables	Total	Q1	Q2	Q3	Q4	P value
CRP (mg/L)	2.55 ± 1.11	4.11 ± 0.68	2.49 ± 0.63	1.93 ± 0.11	1.55 ± 0.11	<0.001
IL-6 (pg/mL)	3.83 ± 1.41	5.41 ± 1.51	3.45 ± 1.31	3.44 ± 0.20	2.87 ± 0.11	<0.001
TNF-α (pg/mL)	5.29 ± 1.94	7.35 ± 1.73	6.12 ± 1.58	4.12 ± 0.20	3.58 ± 0.12	<0.001
**TMAO**	3.94 ± 1.45	5.99 ± 0.89	3.90 ± 0.63	3.11 ± 0.17	2.62 ± 0.13	<0.001
LBP (µg/mL)	18.67 ± 4.60	24.35 ± 4.09	18.67 ± 3.12	16.85 ± 0.88	14.47 ± 0.46	<0.001
Adiponectin (µg/mL)	10.80 ± 1.38	9.76 ± 1.46	10.55 ± 0.99	11.35 ± 0.58	11.58 ± 1.38	<0.001
Leptin (ng/mL)	17.09 ± 2.96	19.41 ± 3.26	17.43 ± 2.25	15.77 ± 1.27	15.65 ± 2.72	<0.001

Values are expressed as mean ± standard deviation. P values were derived from one-way ANOVA. Abbreviations: CRP, C-reactive protein; IL-6, interleukin-6; TNF-α, tumor necrosis factor-alpha; LBP, lipopolysaccharide-binding protein.

Glycemic parameters differed significantly across DPI quartiles (Table 3). Compared to participants in the lowest DPI quartile (Q1, n = 164), those in the highest quartile (Q4, n = 156) exhibited lower mean fasting plasma glucose (4.52 ± 0.06 mmol/L vs. 5.92 ± 0.24 mmol/L; P < 0.001), lower insulin concentrations (65.6 ± 2.4 pmol/L vs. 120.4 ± 18.0 pmol/L; P < 0.001), and lower HOMA-IR values (1.90 ± 0.09 vs. 4.59 ± 0.82; P < 0.001). Mean HbA1c was also lower in Q4 compared to Q1 (35.9 ± 6.2 mmol/mol vs. 43.9 ± 7.3 mmol/mol; P < 0.001).

**Table 3:** Glycemic and Lipid Profile by DPI Quartiles.

Variables	Total	Q1	Q2	Q3	Q4	P value
HbA1c (mmol/mol)	39.0 ± 6.5	43.9 ± 7.3	39.7 ± 4.8	36.3 ± 2.9	35.9 ± 6.2	<0.001
FPG (mmol/L)	5.11 ± 0.58	5.92 ± 0.24	5.23 ± 0.24	4.75 ± 0.07	4.52 ± 0.06	<0.001
Insulin (pmol/L)	87.2 ± 24.3	120.4 ± 18.0	85.9 ± 12.6	74.5 ± 3.2	65.6 ± 2.4	<0.001
HOMA-IR	2.94 ± 1.18	4.59 ± 0.82	2.89 ± 0.53	2.27 ± 0.14	1.90 ± 0.09	<0.001
HDL-C (mmol/L)	1.41 ± 0.18	1.28 ± 0.19	1.39 ± 0.14	1.49 ± 0.08	1.50 ± 0.16	<0.001
LDL-C (mmol/L)	3.01 ± 0.48	3.39 ± 0.50	3.09 ± 0.39	2.80 ± 0.19	2.74 ± 0.44	<0.001
Triglycerides (mmol/L)	1.54 ± 0.16	1.67 ± 0.17	1.56 ± 0.12	1.48 ± 0.06	1.45 ± 0.16	<0.001
Total cholesterol (mmol/L)	5.62 ± 0.54	6.04 ± 0.59	5.68 ± 0.43	5.41 ± 0.22	5.34 ± 0.51	<0.001

Data are expressed as mean ± standard deviation. P values obtained using one-way ANOVA across DPI quartiles.

HOMA-IR = Homeostatic Model Assessment for Insulin Resistance (calculated using glucose in mmol/L and insulin in pmol/L: HOMA-IR = [glucose (mmol/L) × insulin (pmol/L)] / 22.5).

Lipid profiles showed significant variation by DPI quartile. Mean HDL-C concentrations were higher in Q4 than Q1 (1.50 ± 0.16 mmol/L vs. 1.28 ± 0.19 mmol/L; P < 0.001). Conversely, mean LDL-C (2.74 ± 0.44 mmol/L vs. 3.39 ± 0.50 mmol/L; P < 0.001), triglycerides (1.45 ± 0.16 mmol/L vs. 1.67 ± 0.17 mmol/L; P < 0.001), and total cholesterol (5.34 ± 0.51 mmol/L vs. 6.04 ± 0.59 mmol/L; P < 0.001) were lower in Q4 compared to Q1. Tests for linear trend across DPI quartiles were significant for all glycemic and lipid parameters (all P for trend < 0.001).

Multivariable linear regression analyses (Table 4) confirmed that these associations were robust after adjustment for confounders including age, sex, BMI, physical activity, body composition, energy, and fiber intake. Higher DPI quartiles were independently associated with lower inflammatory (CRP, IL-6, TNF-α, TMAO) and metabolic markers (HbA1c, FPG, insulin, HOMA-IR; all P for trend < 0.001).

**Table 4:** Multivariable Linear Regression of DPI Quartiles and Gut Microbiota Diversity, Inflammatory Markers, and Metabolic Parameters.

Variable	DPI Quartile	Crude Model	Model 1	Model 2	p for trend
CRP	Q1	Ref	Ref	Ref	
	Q2	−1.62 (−1.77 to −1.47)	−0.79 (−0.94 to −0.64)	−0.86 (−1.00 to −0.73)	<0.001
	Q3	−2.18 (−2.32 to −2.03)	−0.96 (−1.14 to −0.78)	−1.04 (−1.22 to −0.85)	
	Q4	−2.56 (−2.70 to −2.41)	−0.94 (−1.18 to −0.71)	−0.85 (−1.10 to −0.60)	
IL-6	Q1	Ref	Ref	Ref	
	Q2	−1.95 (−2.28 to −1.62)	−0.44 (−0.76 to −0.11)	−0.85 (−1.11 to −0.59)	<0.001
	Q3	−1.96 (−2.28 to −1.65)	0.32 (−0.07 to 0.71)	−0.77 (−1.13 to −0.40)	
	Q4	−2.54 (−2.85 to −2.22)	0.26 (−0.26 to 0.78)	−0.69 (−1.17 to −0.20)	
TNF-α	Q1	Ref	Ref	Ref	
	Q2	−1.24 (−1.62 to −0.85)	−1.73 (−2.19 to −1.28)	−1.00 (−1.29 to −0.71)	<0.001
	Q3	−3.24 (−3.60 to −2.87)	−4.13 (−4.68 to −3.58)	−1.68 (−2.09 to −1.27)	
	Q4	−3.77 (−4.13 to −3.41)	−4.36 (−5.08 to −3.63)	−1.61 (−2.15 to −1.07)	
TMAO	Q1	Ref	Ref	Ref	
	Q2	−2.09 (−2.28 to −1.91)	−1.13 (−1.32 to −0.94)	−1.18 (−1.36 to −1.00)	<0.001
	Q3	−2.88 (−3.05 to −2.70)	−1.48 (−1.70 to −1.25)	−1.45 (−1.70 to −1.19)	
	Q4	−3.37 (−3.54 to −3.20)	−1.47 (−1.77 to −1.17)	−1.26 (−1.59 to −0.92)	
HbA1c	Q1	Ref	Ref	Ref	
	Q2	−0.38 (−0.55 to −0.22)	−0.22 (−0.45 to 0.01)	−0.23 (−0.46 to −0.00)	<0.001
	Q3	−0.70 (−0.86 to −0.54)	−0.45 (−0.72 to −0.17)	−0.46 (−0.78 to −0.14)	
	Q4	−0.73 (−0.89 to −0.57)	−0.46 (−0.83 to −0.10)	−0.45 (−0.87 to −0.02)	
FPG	Q1	Ref	Ref	Ref	
	Q2	−12.41 (−13.44 to −11.38)	−8.67 (−9.94 to −7.39)	−7.54 (−8.58 to −6.50)	<0.001
	Q3	−21.07 (−22.05 to −20.08)	−15.87 (−17.41 to −14.34)	−10.96 (−12.42 to −9.49)	
	Q4	−25.21 (−26.19 to −24.23)	−17.30 (−19.34 to −15.26)	−10.54 (−12.47 to −8.60)	
Insulin	Q1	Ref	Ref	Ref	
	Q2	−4.96 (−5.49 to −4.43)	−2.25 (−2.80 to −1.71)	−2.64 (−3.11 to −2.17)	<0.001
	Q3	−6.60 (−7.11 to −6.09)	−2.62 (−3.27 to −1.96)	−3.26 (−3.92 to −2.60)	
	Q4	−7.88 (−8.38 to −7.38)	−2.64 (−3.51 to −1.77)	−2.73 (−3.60 to −1.87)	
HOMA-IR	Q1	Ref	Ref	Ref	
	Q2	−1.70 (−1.86 to −1.53)	−0.89 (−1.07 to −0.71)	−0.95 (−1.11 to −0.80)	<0.001
	Q3	−2.32 (−2.47 to −2.16)	−1.14 (−1.35 to −0.92)	−1.15 (−1.38 to −0.93)	
	Q4	−2.69 (−2.84 to −2.53)	−1.10 (−1.38 to −0.81)	−0.92 (−1.21 to −0.62)	

Values represent β coefficients and 95% confidence intervals (CIs) from linear regression analyses comparing quartiles of the DPI, with Quartile 1 (Q1) as the reference. P-trend values were calculated using linear regression with DPI as a continuous variable to assess the linear trend of the association with each outcome variable. Crude model is unadjusted. Model 1 adjusts for age, sex, and body mass index (BMI). Model 2 further adjusts for physical activity (MET), fat mass, muscle mass, energy intake (kcal/day), and fiber intake (g/day). Abbreviations: CRP = C-reactive protein; IL-6 = Interleukin-6; TNF-α = Tumor necrosis factor-alpha; TMAO = Trimethylamine N-oxide; HbA1c = Glycated hemoglobin; FPG = Fasting blood sugar; HOMA-IR = Homeostasis model assessment of insulin resistance.

Collectively, these results suggest that higher dietary phytochemical intake is associated with improved anthropometric measures, favorable metabolic and lipid profiles, reduced systemic inflammation, and enhanced gut metabolites, with the latter partially mediating the beneficial metabolic and inflammatory effects of a phytochemical-rich diet.

## Discussion

Our initial hypothesis that higher adherence to a phytochemical-rich diet improves metabolic health by modulating gut metabolites and reducing systemic inflammation was confirmed by our findings. This cross-sectional study demonstrated that higher adherence to a phytochemical-rich dietary pattern, as indicated by a higher DPI, was significantly associated with favorable anthropometric, metabolic, and inflammatory markers among adults with obesity. Participants in the highest DPI quartile exhibited lower BMI, waist circumference, and fat mass, alongside improved glycemic control, lipid profile, and systemic inflammation markers, including CRP, IL-6, TNF-α, and LBP. Notably, higher DPI was also linked to elevated adiponectin levels, and lower plasma TMAO concentrations. These associations are consistent with the hypothesis that dietary phytochemicals may influence host metabolism in conjunction with gut metabolites ecology and reduced inflammation, though causal relationships cannot be established from this cross-sectional design.

Our findings align with previous research demonstrating that plant-based and antioxidant-rich dietary patterns, such as the Mediterranean, MIND, and DASH diets, are inversely associated with inflammatory and metabolic disturbances in obesity [16–18]. Similar to our results, studies utilizing the Dietary Inflammatory Index (DII) have shown that lower DII scores—reflecting anti-inflammatory dietary patterns—correlate with reduced CRP, IL-6, and TNF-α levels, as well as improved insulin sensitivity and lipid metabolism [9,19–21]. However, while the DII primarily quantifies the inflammatory potential of nutrients, the DPI captures the overall contribution of phytochemical-rich foods to total energy intake, encompassing a broader spectrum of bioactive compounds such as flavonoids, carotenoids, phenolic acids, and lignans [8]. These compounds exert antioxidant and anti-inflammatory effects by modulating transcription factors (e.g., NF-κB, Nrf2), cytokine production, and endothelial function, which may collectively mitigate metabolic inflammation [9,12]. More recent applications of the DPI have demonstrated consistent associations with cardiometabolic risk factors across diverse populations [14,22,23], supporting its utility as a dietary quality indicator. The observed multidimensional benefits of higher DPI highlight its potential utility as a simple, food-based index reflecting both dietary quality and metabolic resilience in obesity.

An important contribution of this study lies in linking dietary phytochemical intake with gut metabolites (TMAO) metabolism. Consistent with our observations, previous reports have shown that diets abundant in fruits, vegetables, and whole grains increase alpha diversity and beneficial taxa such as Bifidobacterium and Faecalibacterium, while reducing the Firmicutes/Bacteroidetes ratio—an imbalance often associated with obesity and insulin resistance [24]. Increased microbial diversity has been implicated in enhanced production of short-chain fatty acids (SCFAs), particularly butyrate, which improves gut barrier integrity, reduces endotoxemia, and attenuates systemic inflammation [25]. Conversely, lower microbial diversity and higher F/B ratios favor metabolic endotoxemia and TMAO generation, both contributing to insulin resistance [5,26]. Our data support this mechanistic pathway, as higher DPI was associated with lower circulating TMAO and LBP levels, suggesting improved intestinal integrity and reduced microbial-derived inflammation. However, we cannot exclude the possibility that lower TMAO levels may also reflect reduced intake of TMAO precursors themselves (e.g., choline and carnitine from animal products), as individuals with higher DPI consumed less total energy and had lower protein and fat intake (Table 1). Future studies should measure dietary choline and carnitine intake to disentangle these effects [5]. Unlike previous DPI studies that focused primarily on inflammatory or cardiometabolic markers, our inclusion of TMAO adds mechanistic insight into phytochemical–microbiome–metabolic interactions. These results collectively underscore the interconnection between dietary phytochemical exposure, microbial-derived metabolites, and systemic inflammatory tone in obesity pathophysiology.

Previous experimental studies have shown that polyphenols such as quercetin, resveratrol, and catechins modulate gut microbial composition, suppress pathogenic bacteria, and promote SCFA-producing commensals. These microbiota-related effects may contribute to improved glucose homeostasis and lipid metabolism, partly explaining the inverse association between DPI and HOMA-IR observed in this study [27,28]. Such findings emphasize the potential role of gut metabolites as a therapeutic target for enhancing the metabolic efficacy of phytochemical-rich dietary interventions. It is important to note that the mediation analysis does not prove causation but rather identifies statistical relationships consistent with a hypothesized pathway. The observed associations could alternatively reflect: (1) direct effects of phytochemicals on metabolic outcomes independent of microbiota; (2) effects of improved metabolic health on dietary choices and microbiota composition; or (3) confounding by lifestyle factors associated with both diet quality and metabolic health. These possibilities cannot be distinguished in the current cross-sectional design. In conclusion, the results should be interpreted cautiously, as temporal sequencing between diet, microbiota, inflammation, and insulin resistance cannot be established. We further emphasized that the observed associations may reflect multiple biological and behavioral pathways and cannot confirm mechanistic relationships

The strengths of this study include a relatively large, well-characterized cohort of adults with obesity, comprehensive biochemical profiling, and integration of dietary, microbial, and inflammatory data within a single model. The use of validated assessment tools, standardized 16S rRNA sequencing, and adjustment for potential confounders enhances the robustness of the findings. Additionally, the simultaneous evaluation of TMAO provides novel insight into diet–microbiota–metabolism interactions in obesity. Nevertheless, several limitations should be acknowledged. First, the cross-sectional design precludes causal inference; mediation analysis cannot establish temporal precedence or rule out reverse causality. Second, although the FFQ was validated in the study population, it is subject to recall bias and may not capture seasonal variations in phytochemical-rich food consumption or accurately estimate portion sizes. Third, the DPI treats all phytochemical-rich foods equally and does not account for variability in phytochemical density within food groups (e.g., berries versus apples; dark leafy greens versus cucumbers), nor does it differentiate among phytochemical subclasses (flavonoids, carotenoids, phenolic acids) that may have distinct biological activities. This restricts our ability to infer metabolic pathways that might mediate the observed associations, such as short-chain fatty acid production or bile acid metabolism [22]. The absence of fecal metabolomic or metagenomic profiling means we cannot directly link specific microbial functions to metabolic outcomes. Future studies incorporating shotgun metagenomic sequencing and targeted metabolomics would enable more definitive mechanistic insights. Furthermore, despite covariate adjustment, residual confounding by unmeasured factors remains possible. We lacked data on socioeconomic status, sleep, stress, and overall dietary patterns beyond DPI—factors that may influence both diet and metabolic outcomes [29]. Physical activity was assessed via self-report (IPAQ), subject to recall bias. Future prospective and interventional studies are warranted to confirm causal pathways. Such studies should employ multiple 24-hour recalls or food records combined with biomarker-based assessments of phytochemical intake (e.g., urinary polyphenol metabolites) to provide more precise exposure estimates, and elucidate whether phytochemical-rich dietary interventions can modulate gut microbiota and improve insulin sensitivity in individuals with obesity.

## Conclusions

In this cross-sectional study of adults with obesity, higher DPI **was associated** with more favorable profiles of systemic inflammation, insulin sensitivity, and circulating TMAO. These findings identify associations consistent with hypothesized pathways linking plant-based dietary patterns to metabolic health, though causal relationships cannot be inferred from this cross-sectional design. Prospective cohort studies and randomized controlled trials are needed to determine whether increasing phytochemical-rich food consumption can modify gut microbiota composition and improve metabolic outcomes in individuals with obesity

## Supporting information

S1 File STROBE checklist. (DOCX)

S2 File Raw data. (XLSX)
