# Supplementary material for: Dietary phytochemical index is associated with systemic inflammation, insulin resistance, and gut microbiota-derived metabolites in individuals with obesity: A cross-sectional study
Source: PLoS One. 2026 Apr 27;21(4):e0347754. doi: 10.1371/journal.pone.0347754 (PMC13119903; doi:10.1371/journal.pone.0347754)
Supplement: S1 File — (DOCX) [file pone.0347754.s001.docx]

**STrengthening the Reporting of OBservational studies in Epidemiology**.

|  |  | Reporting Item | Page Number |
| --- | --- | --- | --- |
| **Title and abstract** |  |  |  |
| Title | [#1a](https://www.goodreports.org/reporting-checklists/strobe-case-control/info/#1a) | Indicate the study’s design with a commonly used term in the title or the abstract | **Page 1, Line 1–3** (“Cross-sectional Study”) |
| Abstract | [#1b](https://www.goodreports.org/reporting-checklists/strobe-case-control/info/#1b) | Provide in the abstract an informative and balanced summary of what was done and what was found | **Page 1, Lines 4–68** |
| **Introduction** |  |  |  |
| Background / rationale | [#2](https://www.goodreports.org/reporting-checklists/strobe-case-control/info/#2) | Explain the scientific background and rationale for the investigation being reported | **Page 2–3, Lines 69–150** |
| Objectives | [#3](https://www.goodreports.org/reporting-checklists/strobe-case-control/info/#3) | State specific objectives, including any prespecified hypotheses | **Page 3, Lines 147–150** |
| **Methods** |  |  |  |
| Study design | [#4](https://www.goodreports.org/reporting-checklists/strobe-case-control/info/#4) | Present key elements of study design early in the paper | **Page 3–4, Lines 152–185** |
| Setting | [#5](https://www.goodreports.org/reporting-checklists/strobe-case-control/info/#5) | Describe the setting, locations, and relevant dates, including periods of recruitment, exposure, follow-up, and data collection | **Page 4, Lines 152–174** |
| Eligibility criteria | [#6a](https://www.goodreports.org/reporting-checklists/strobe-case-control/info/#6a) | Give the eligibility criteria, and the sources and methods of case ascertainment and control selection. Give the rationale for the choice of cases and controls. For matched studies, give matching criteria and the number of controls per case | **Page 4, Lines 160–180** |
| Eligibility criteria | [#6b](https://www.goodreports.org/reporting-checklists/strobe-case-control/info/#6b) | For matched studies, give matching criteria and the number of controls per case | **NA** |
|  | [#7](https://www.goodreports.org/reporting-checklists/strobe-case-control/info/#7) | Clearly define all outcomes, exposures, predictors, potential confounders, and effect modifiers. Give diagnostic criteria, if applicable | **All variables defined in Methods pages 4–6** *(but no central “variable definitions” table)* → You marked NA. Correct = **Page 4–6, Lines 174–260** |
| Data sources / measurement | [#8](https://www.goodreports.org/reporting-checklists/strobe-case-control/info/#8) | For each variable of interest give sources of data and details of methods of assessment (measurement). Describe comparability of assessment methods if there is more than one group. Give information separately for cases and controls. | **Page 4–6, Lines 174–260** |
| Bias | [#9](https://www.goodreports.org/reporting-checklists/strobe-case-control/info/#9) | Describe any efforts to address potential sources of bias | **Page 12, Lines ~750–770 (Limitations section)** |
| Study size | [#10](https://www.goodreports.org/reporting-checklists/strobe-case-control/info/#10) | Explain how the study size was arrived at | **Page 6, Lines 258–270 (Sample size via G*Power)** |
| Quantitative variables | [#11](https://www.goodreports.org/reporting-checklists/strobe-case-control/info/#11) | Explain how quantitative variables were handled in the analyses. If applicable, describe which groupings were chosen, and why | **Page 6, Lines 247–260** |
| Statistical methods | [#12a](https://www.goodreports.org/reporting-checklists/strobe-case-control/info/#12a) | Describe all statistical methods, including those used to control for confounding | **Page 6–7, Lines 247–310** |
| Statistical methods | [#12b](https://www.goodreports.org/reporting-checklists/strobe-case-control/info/#12b) | Describe any methods used to examine subgroups and interactions | **Page 7, Lines 290–310** (mediation & subgroup analyses) |
| Statistical methods | [#12c](https://www.goodreports.org/reporting-checklists/strobe-case-control/info/#12c) | Explain how missing data were addressed | **Page 7, Lines 290–299** *(missing data exclusion in analysis)* |
| Statistical methods | [#12d](https://www.goodreports.org/reporting-checklists/strobe-case-control/info/#12d) | If applicable, explain how matching of cases and controls was addressed | **NA** |
| Statistical methods | [#12e](https://www.goodreports.org/reporting-checklists/strobe-case-control/info/#12e) | Describe any sensitivity analyses | **NA** |
| **Results** |  |  |  |
| Participants | [#13a](https://www.goodreports.org/reporting-checklists/strobe-case-control/info/#13a) | Report numbers of individuals at each stage of study—eg numbers potentially eligible, examined for eligibility, confirmed eligible, included in the study, completing follow-up, and analysed. Give information separately for cases and controls. | Page 8, Lines ~328–350 & Figure 1 |
| Participants | [#13b](https://www.goodreports.org/reporting-checklists/strobe-case-control/info/#13b) | Give reasons for non-participation at each stage | Page 8 & Figure 1 |
| Participants | [#13c](https://www.goodreports.org/reporting-checklists/strobe-case-control/info/#13c) | Consider use of a flow diagram | Figure 1 included (Page 8) |
| Descriptive data | [#14a](https://www.goodreports.org/reporting-checklists/strobe-case-control/info/#14a) | Give characteristics of study participants (eg demographic, clinical, social) and information on exposures and potential confounders. Give information separately for cases and controls | Page 8–9, Table 1 |
| Descriptive data | [#14b](https://www.goodreports.org/reporting-checklists/strobe-case-control/info/#14b) | Indicate number of participants with missing data for each variable of interest | Page 8, Table 1 (no missing reported) |
| Outcome data | [#15](https://www.goodreports.org/reporting-checklists/strobe-case-control/info/#15) | Report numbers in each exposure category, or summary measures of exposure. Give information separately for cases and controls | Page 8–10, Tables 1–3 |
| Main results | [#16a](https://www.goodreports.org/reporting-checklists/strobe-case-control/info/#16a) | Give unadjusted estimates and, if applicable, confounder-adjusted estimates and their precision (eg, 95% confidence interval). Make clear which confounders were adjusted for and why they were included | Page 9–10, Table 4 |
| Main results | [#16b](https://www.goodreports.org/reporting-checklists/strobe-case-control/info/#16b) | Report category boundaries when continuous variables were categorized | Page 8–9 (quartiles of DPI defined in tables) |
| Main results | [#16c](https://www.goodreports.org/reporting-checklists/strobe-case-control/info/#16c) | If relevant, consider translating estimates of relative risk into absolute risk for a meaningful time period |  |
| Other analyses | [#17](https://www.goodreports.org/reporting-checklists/strobe-case-control/info/#17) | Report other analyses done—e.g., analyses of subgroups and interactions, and sensitivity analyses | Page 10, Table 5 + mediation (Figure 2) |
| **Discussion** |  |  |  |
| Key results | [#18](https://www.goodreports.org/reporting-checklists/strobe-case-control/info/#18) | Summarise key results with reference to study objectives | Page 10–11, Lines ~520–580 |
| Limitations | [#19](https://www.goodreports.org/reporting-checklists/strobe-case-control/info/#19) | Discuss limitations of the study, taking into account sources of potential bias or imprecision. Discuss both direction and magnitude of any potential bias. | Page 12, Lines ~750–790 |
| Interpretation | [#20](https://www.goodreports.org/reporting-checklists/strobe-case-control/info/#20) | Give a cautious overall interpretation considering objectives, limitations, multiplicity of analyses, results from similar studies, and other relevant evidence. | Page 11–13 |
| Generalisability | [#21](https://www.goodreports.org/reporting-checklists/strobe-case-control/info/#21) | Discuss the generalisability (external validity) of the study results | Page 13, Lines ~800–820 |
| **Other Information** |  |  |  |
| Funding | [#22](https://www.goodreports.org/reporting-checklists/strobe-case-control/info/#22) | Give the source of funding and the role of the funders for the present study and, if applicable, for the original study on which the present article is based | Page 13–14, Lines ~840–900 (Funding, Ethics, Declarations) |

None The STROBE checklist is distributed under the terms of the Creative Commons Attribution License CC-BY. This checklist can be completed online using <https://www.goodreports.org/>, a tool made by the [EQUATOR Network](https://www.equator-network.org) in collaboration with [Penelope.ai](https://www.penelope.ai)
